# Variability in Avian Eggshell Colour: A Comparative Study of Museum Eggshells

**DOI:** 10.1371/journal.pone.0012054

**Published:** 2010-08-09

**Authors:** Phillip Cassey, Steven J. Portugal, Golo Maurer, John G. Ewen, Rebecca L. Boulton, Mark E. Hauber, Tim M. Blackburn

**Affiliations:** 1 Centre for Ornithology, School of Biosciences, University of Birmingham, Birmingham, United Kingdom; 2 Institute of Zoology, Zoological Society of London, London, United Kingdom; 3 Department of Psychology, Hunter College of the City University of New York, New York, New York, United States of America; Field Museum of Natural History, United States of America

## Abstract

**Background:**

The exceptional diversity of coloration found in avian eggshells has long fascinated biologists and inspired a broad range of adaptive hypotheses to explain its evolution. Three main impediments to understanding the variability of eggshell appearance are: (1) the reliable quantification of the variation in eggshell colours; (2) its perception by birds themselves, and (3) its relation to avian phylogeny. Here we use an extensive museum collection to address these problems directly, and to test how diversity in eggshell coloration is distributed among different phylogenetic levels of the class Aves.

**Methodology and Results:**

Spectrophotometric data on eggshell coloration were collected from a taxonomically representative sample of 251 bird species to determine the change in reflectance across different wavelengths and the taxonomic level where the variation resides. As many hypotheses for the evolution of eggshell coloration assume that egg colours provide a communication signal for an avian receiver, we also modelled reflectance spectra of shell coloration for the avian visual system. We found that a majority of species have eggs with similar background colour (long wavelengths) but that striking differences are just as likely to occur between congeners as between members of different families. The region of greatest variability in eggshell colour among closely related species coincided with the medium-wavelength sensitive region around 500 nm.

**Conclusions:**

The majority of bird species share similar background eggshell colours, while the greatest variability among species aligns with differences along a red-brown to blue axis that most likely corresponds with variation in the presence and concentration of two tetrapyrrole pigments responsible for eggshell coloration. Additionally, our results confirm previous findings of temporal changes in museum collections, and this will be of particular concern for studies testing intraspecific hypotheses relating temporal patterns to adaptation of eggshell colour. We suggest that future studies investigating the phylogenetic association between the composition and concentration of eggshell pigments, and between the evolutionary drivers and functional impacts of eggshell colour variability will be most rewarding.

## Introduction

**Figure 1:**
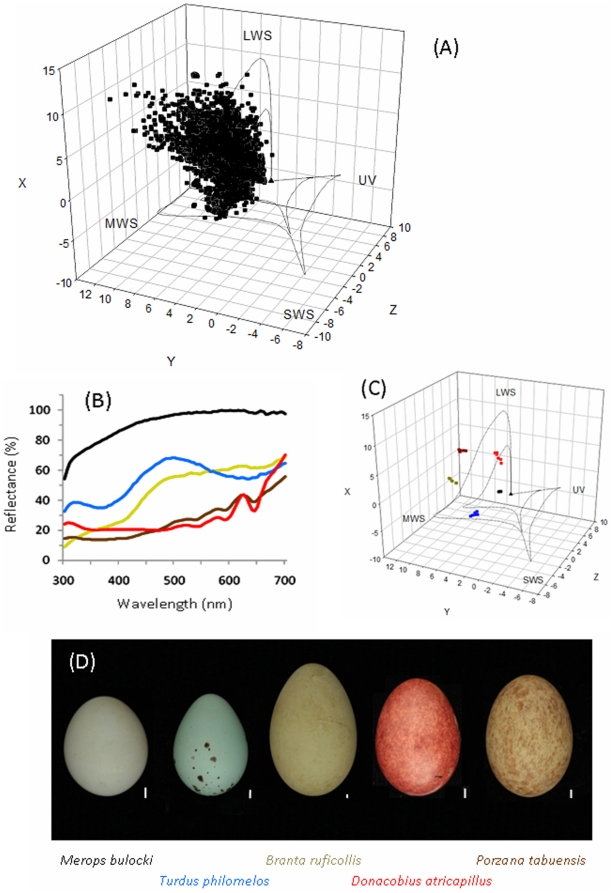
Avian eggshell colours. (A) 3D diagram for all spectra (see methods) in an avian tetrachromatic colour opponent space following Kelber et al. [33]. Boundaries are drawn following Cassey et al. [12] and labels indicate whether colours reflect maximally in the ultraviolet- (UV), short- (SWS), medium- (MWS), or long-wavelength sensitive regions of the spectrum. (B) Average reflectance spectra for five representative eggs as represented in (C) for their replicate (n = 6) individual reflectance spectra plotted in the same 3D tetrachromatic space. (D) The eggs of the five representative avian species as photographed courtesy of the Natural History Museum, Tring, United Kingdom. Colours of the lines in (B) and the loci in (C) correspond to the text colours of the species labels in (D).

Colourful biological pigments are among the most conspicuous products of metabolism and serve a wide variety of physical, physiological, and behavioural functions [1]. Accurate descriptions of diverse pigmentation, and the resulting coloration, are therefore of fundamental interest to evolutionary biologists and behavioural ecologists alike [2]. One of the most fascinating examples of biological pigmentation is the variation in the colourful appearance of avian eggshells (Figure 1). The remarkable diversity in eggshell colours and patterns has long intrigued researchers [3], [4], and continues to attract both scientific [5], [6] and popular attention [7]. Eggshell pigmentation is likely to be a key component of the avian reproductive system for two reasons. First, despite the immense interspecific variation in ecology and life-history, birds are surprisingly conservative in their mode of reproduction. Without exception, birds rely on a period of external egg incubation in the course of their reproduction [8]. Second, remarkably, birds alone among vertebrates have evolved pigmentation of their outer shell layer. Despite an increasing interest in the evolutionary drivers of eggshell coloration and maculation [9], little attempt has been made to relate variation in egg coloration to phylogeny or quantify the variability in eggshell colour within versus among diverse taxa.

Previous comparative analyses of eggshell colour have been limited both in the scope of the lineages included [10]–[12], and in the manner coloration was assessed [13]. The most comprehensive analysis of eggshell colour in birds, to date (4417 species sampled), was based on descriptive observations of eggshell colour as perceived by humans [6]. Furthermore, this study assessed likely adaptive functions of egg colours based on ‘typical’ eggshell traits at the level of avian families. Yet, eggshell coloration can vary substantially within avian families [5] and may strongly covary with ecologically relevant selective factors across a variety of taxonomic levels. For example, the family Muscicapidae includes species with highly varied eggshell colours when assessed by a combination of physical and perceptual methods [12]. Moreover, birds with tetrachromatic vision are predicted to discriminate smaller differences in eggshell colour than humans, which are trichromats. This has been shown using colour opponent threshold models [12]. Thus categorical human assessment of colour is likely to underestimate the true functional variability in eggshell appearance, especially when it is based on photographic plates and field guide descriptions.

Here, we employ a comparative framework, implementing a phylogenetically informed statistical approach, and analyse an extensive dataset of avian eggshell samples to quantify the extent to which metrics of eggshell colour are evolutionarily conserved across levels of varying biological organization. We employed a portable reflectance spectrophotometer [14] to measure eggshell spectra over the avian visible range (i.e. 300–700 nm), and used this to produce a range of quantitative measures of background eggshell colour for a large sample of museum specimens from across avian phylogenetic lineages. We then applied methods based on both taxonomic and phylogenetic information to assess the extent to which closely related bird species share eggshell coloration. Following previous analyses [6] we predict that (1) variability in eggshell colour will be phylogenetically labile, and (2) that individual components of coloration will covary among species in different ways at different levels of relatedness. Finally, we considered how eggshell colour varies with time in museum storage. It is expected that this analytical framework will prove useful for biologists studying the variability in pigment adaptation, and eggshell coloration in particular.

## Methods

### Eggshell samples

Clutches from 251 species (2190 eggs) were measured with kind permission of the Natural History Museum (NHM) at Tring, United Kingdom (NHM accession numbers are available from PC on request). Although the nests and eggs of about one third of the world's species may still be undiscovered or undescribed [15], the NHM collection is believed to be the most comprehensive in the world with an estimated *c*. 1 million eggs [16], [17]. Depending on the samples available in the collection, up to five clutches of each species were measured, and up to five eggs from each clutch.

The 251 species were selected using a randomisation program to sort the *c*. 10,000 species in the global avian taxonomy *sensu* Sibley & Monroe [18], with the first 251 species on the sorted list forming the initial sample. Species were equally weighted, and so higher taxon representation was retained and species were chosen (without replacement) with likelihoods proportional to the diversity of their higher taxa. Not all of the 251 species on our sorted list were represented in the NHM collection, although the majority of genera (∼90%) were. In cases where the species were missing we returned to the original unsorted taxonomy and selected the nearest relative available in the collection, in random order (up or down the printed list).

The 251 species we sampled included representatives from 60 (∼40%) bird families, based on the taxonomy of Sibley & Monroe [18]. To assess whether these were a biased sample of all possible species with respect to aspects of species biology, we compared the median adult body mass (from data collated by Dunning [19]) and median breeding range latitudinal midpoint (from data collected by Orme et al. [20]) of the 251 species, with the distribution of 1000 medians for 251 randomly chosen species. These two traits are well suited as surrogates for the life history and geographical variability among bird species [21]. The observed median adult body mass (37.6 g) and breeding range midpoint (−1.16° latitude; latitudes south of the equator were scored as negative) for the 251 species were both included within the range encompassed by 95% of randomly chosen median values (adult body mass = 29.68–48.80 g; breeding range midpoint = −2.76–1.45° latitude). We conclude that our 251 species are an unbiased random sample of the global avifauna, at least with respect to phylogeny, body mass and latitudinal distribution.

The distribution of egg collection dates of our sampled clutches ranged from 1825 to 2002. The median date was 1909 and the decade of highest proportion of collection (113 clutches) was 1901–1910. Based on previous work [22], we analysed the effect of time since collection on the luminance and shape of eggshell reflectance spectra (see below). These two metrics were chosen *a priori* to be the most likely inclusive of effects accrued through museum storage across the variety of different species, eggshell types, and eggshell colours. Where possible, we identified the two clutches in our sample separated by the largest period of time between collections for each species. We then calculated the average difference in luminance and the absolute summed difference between the relative spectra for all eggs in these two clutches. We analysed whether these differences were associated with the length of time between collections using generalised linear models in SAS v9.2 (SAS Institute Inc., Cary, NC, USA).

### Background eggshell colour

Eggshell reflectance was measured *in situ* at the NHM using an Ocean Optics USB2000 Miniature Fiber Optic Spectrophotometer with illumination by a DT mini-lamp. A custom built light-proof cap was fitted over the probe to maintain a consistent angle (90°) between the eggshell and the measuring fibre optics. Spectra were recorded in ∼0.4 nm steps and were expressed relative to a white Ocean Optics WS-1 diffuse reflectance standard. Six measurements were taken from the background shell colour; two in each hemisphere of the eggshell and two at the equator. Considerable care was taken to identify and measure background eggshell colour (as opposed to maculation) in all cases to the best of our ability. To minimize instrument error, dark and white standard reflectance calibration measures were taken regularly during sampling.

We scored the average degree of eggshell maculation from photographs of all the specimens. Eggs were photographed using a Canon EOS 450D digital camera with either a 105 mm or 50 mm Sigma AF lens, depending on egg size. The camera was mounted on a Kaiser camera stand enclosed within two Calumet photographic umbrellas with silver-white (AU3046) and flat white (AU3045) lining. Samples were lit with two OSRAM 11 W energy saving light bulbs producing a light of a colour temperature of 6000 K to the right and front of the sample. The photos were taken at ISO 400 and aperture of f16, while exposure varied from 0.2–6.0 sec depending on the species. For each species, the eggs were assessed by two independent observers for presence and coverage of maculation using a three point scoring system, similar to Kilner [6]. Maculation was recorded for each egg as 0- if the egg was immaculate, 1 - for maculation present but with a clear, dominant background colour, and 2 - for widespread maculation that covered the majority of the egg. An average score was calculated across observers and was highly repeatable (n = 251, r = 0.984). We predict that for immaculate (i.e. non patterned) eggs it will be easier to measure background colour and likely produce more repeatable (less variable) replicate spectra within an egg.

Birds rely heavily on vision for collecting perceptual information from the environment [23], and have some of the most complex retinae of any vertebrate [24]. The avian eye is well evolved for colour discrimination [25], with four spectrally distinct types of single cone photoreceptors [26]. Given the wide taxonomic coverage of species sampled and the limited number of avian microspectrophotometric studies for spectral absorption properties of visual pigments [27], we adopted a conservative approach to implementing perceptual modelling so that the spectral sensitivities of the avian eye was not constrained by assuming the identity and sensory range of the specific receiver for which eggshell colour functions.

Reflection curves were truncated between 300 and 700 nm [12]. An interpolated average was used to calculate an average reflectance value at 5 nm steps. The absolute sum difference (in area) between two relative spectra was calculated by dividing each 5 nm value by the sum of the reflectance curve, subtracting one spectrum from the other, and then summing the absolute differences across all wavelengths. All analyses were conducted in SAS v9.2 (SAS Institute Inc., Cary, NC, USA).

Vertebrate luminance mechanisms tend to use photoreceptors with λ_max_>500 nm [28]. It is most likely that birds use double cones (which contain the LWS pigment) for achromatic (luminance) tasks [29]. Luminance was calculated as the sum of the reflectance curve that corresponded to the avian double cone region of the wavelength (Σλ_500–700_). We note that this measure is highly correlated, across species, with the total area under the reflectance curve or ‘brightness’ *sensu* Montgomerie [30] (n = 251 species, Pearson's *r* = 0.986).

For visual comparison of the variety of eggshell colour stimuli sampled, we constructed a 3D-tetrachromatic conceptual diagram of the individual chromatic stimuli for each reflectance spectrum (see Figure 1), using the full spectral sensitivities for the ‘average’ ultraviolet-sensitive avian eye as tabulated by Endler & Mielke [31]. The violet-sensitive type eye is still very sensitive to UV; it just has relatively less UV sensitivity than the ultraviolet-sensitive type bird eye [31]. A number of different approaches have been proposed to model the tetrachromatic colour space of avian visually relevant colour perception [31]–[34]. We have chosen to follow the methods given in Kelber *et al*. [33] where eggshell colour loci are independent of the stimulus luminance, and Euclidean distance corresponds to hypothetical perceptual differences between eggshell colours [35].

We reiterate that for comparative purposes, given that we are not making any specific assumptions about the identity or the role of the perceptual receiver of the stimuli, it is not unreasonable to use a single average avian visual model for demonstrating the tetrachromatic space in which eggshell colour signals might hypothetically lie. It is apparent that both photoreceptor spectral sensitivities and photoreceptor densities are conservative, with little evidence for adaptive or systematic variation across a wide variety of species [26], [36], [37]. In addition, all eyes are constrained by the same fundamental problems that limit sensitivity and spatial resolution [23].

### Taxonomic and phylogenetic analysis

The details of the avian phylogeny are contentious, especially in terms of the relative branching positions of higher taxa [38]–[41]. The taxonomic distribution of species amongst higher taxa, however, is much less controversial. Therefore, we used a combination of phylogenetic and taxonomic approaches to assess the extent to which the eggshells of closely related bird species differ in their interspecific coloration. Details of our phylogeny and the phylogenetic hypothesis are provided in Text S1, Text S2, and Figure S1.

First, we calculated the summed absolute differences in the average relative eggshell spectra for each of 107 independent pairs of sister species contained in our putative phylogeny. We defined sister species here as independent pairs of species separated by a range of taxonomic distances: 25 of the comparisons were between species in the same genus, 19 between species in the same tribe (but different genera), 14 between species in the same subfamily (but different tribes), 44 species in the same family (but different subfamilies), and 5 species between different families. We compared the values of the relative spectral differences across these different taxonomic distance categories using one-way analysis of variance.

Second, we assessed how variation in the reflectance spectra partitioned out across avian taxonomic levels using variance components analysis. Nested analysis of variance (PROC NESTED; SAS Institute Inc., Cary, NC, USA) was conducted across the wavelength for each 5 nm interpolated average to assess how variation was distributed (1) among the six replicate measurements within an egg, (2) among eggs within a clutch, (3) among clutches within a species, (4) among species within a family, and (5) among families. We limited higher-level comparisons to families as the classification of species to these groupings is relatively stable.

Third, we calculated the maximum likelihood value of Pagel's λ [42] for luminance as well as each of the independent X, Y, Z tetrachromatic co-ordinates for spectral sensitivity of eggshell coloration. Pagel's λ is a multiplier of the off-diagonal elements of the covariance matrix that quantifies the degree of phylogenetic relatedness between species. Pagel's λ was calculated in R version 2.8 using the APE (Analysis of Phylogenetics and Evolution) package [43] and code written by RP Duncan (Lincoln University, New Zealand). Pagel's λ = 0 indicates that values of a trait are independent of phylogeny, while Pagel's λ = 1 indicates that traits are evolving according to Brownian motion on the given phylogeny. Intermediate values of Pagel's λ imply that traits have evolved according to a process in which the effect of phylogeny is weaker than in the Brownian model [44]. We tested whether each maximum likelihood value of Pagel's λ was significantly different from either 0 or 1 by comparing the log-likelihood values for luminance as well as each of the four regions of spectral sensitivity using likelihood ratio tests, as described by Freckleton et al. [44].

## Results

Variability in eggshell colour between bird species can be obvious to the human eye (Figure 1). Yet, for the majority of species sampled (88%), average eggshell reflectance was greatest in a single region of the spectrum, *sensu* Endler & Mielke [31]; the long-wavelength sensitive region. Eggshells of all of the remaining species reflected maximally in the medium-wavelength sensitive region. In a conceptual model of predicted avian tetrachromatic colour space, the coordinates of the median eggshell reflectance were: X = −2.16, Y = 2.66, Z = 3.13 (Figure 1A), and 58.8% of species had at least one reflectance locus that lay within 1 Euclidean unit (Δ*e*; just-noticeable-difference: JND) of the median.

It was predicted that long-term storage of eggs within the museum would affect both the luminance of the reflected spectra and their overall shape. For species in which reliable collection dates of multiple clutches could be ascertained (43%) there was a tendency (not statistically significant; α = 0.05) for museum clutches collected more recently to have larger values of luminance (paired t-test; t = 1.82, n = 108, P = 0.071). The difference in years (log transformed), between clutches of the same species, was also not significantly related to changes in luminance (estimate ± std err = 0.323±0.486, t = 0.66, n = 108, P = 0.508). The difference in years (log transformed), between clutches of the same species, was, however, positively related to larger absolute sum differences between spectra (estimate ± std err = 0.004±0.002, t = 2.51, n = 108, P = 0.014).

**Figure 2:**
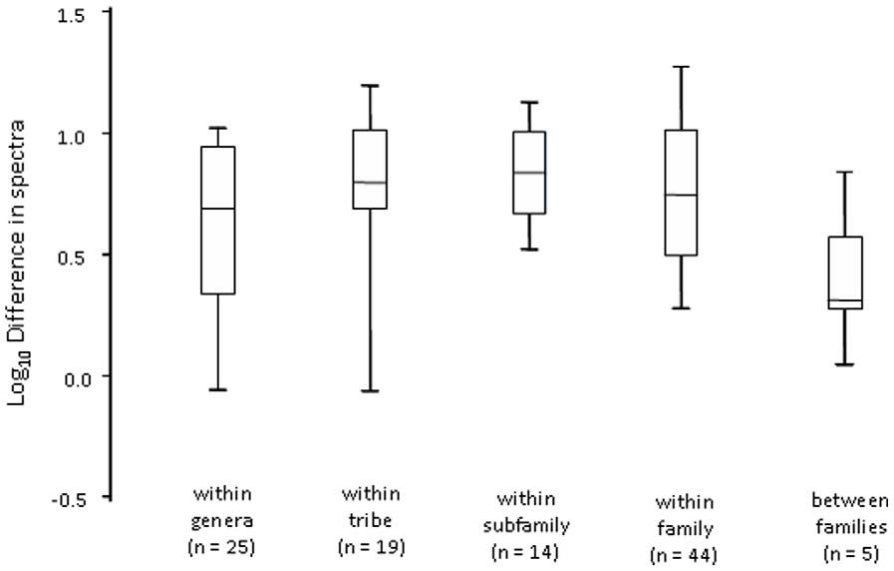
Taxonomic differences in relative reflectance spectra between sister species. Boxplots (median, lower and upper quartiles, and one standard deviation below and above the mean) of the sum differences between reflectance spectra of sister taxa from varying taxonomic levels.

There was no significant variation in the sum difference of average relative reflectance spectra between sister species in our phylogeny from different taxonomic levels (Figure 2; *F*_4,102_ = 1.59, *P* = 0.182). Thus, the degree to which two species were related was not associated with the similarity of their average relative reflectance spectra. Over half (53%) of the largest median differences between the average relative reflectance spectra (of sister species) were in the wavelength interval between 400 and 500 nm.

**Figure 3:**
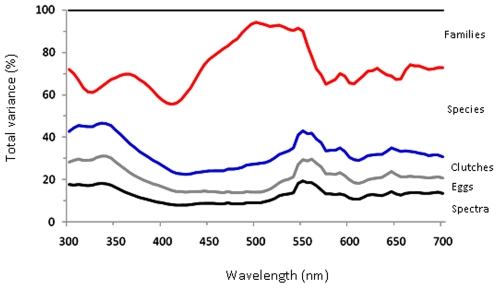
Taxonomic variability in the percentage reflectance of eggshell spectra. Results from nested analysis of variance (nested ANOVA), at 5 nm steps across the wavelength. Coloured lines indicate the cumulative percentage of the variability that occurs between replicate measures within an egg (black line), between different eggs within a clutch (grey line), between different clutches within a species (blue line), between different species within a family (red line), and between different families (top black line).

The percentage of variability in reflectance of eggshell colour spectra accounted for by taxonomy differed across the wavelength, and this was most apparent at short and long wavelengths (Figure 3). Across the wavelength, the percentage of total variability in eggshell reflectance among the six repeated spectra was always less than 20% (average = 12.5%) (Figure 3). The greatest proportion of variance, between higher taxonomic levels, occurred at around 420 nm where differences between families accounted for over 40% of the total variation in spectral reflectance (Figure 3).

**Figure 4:**
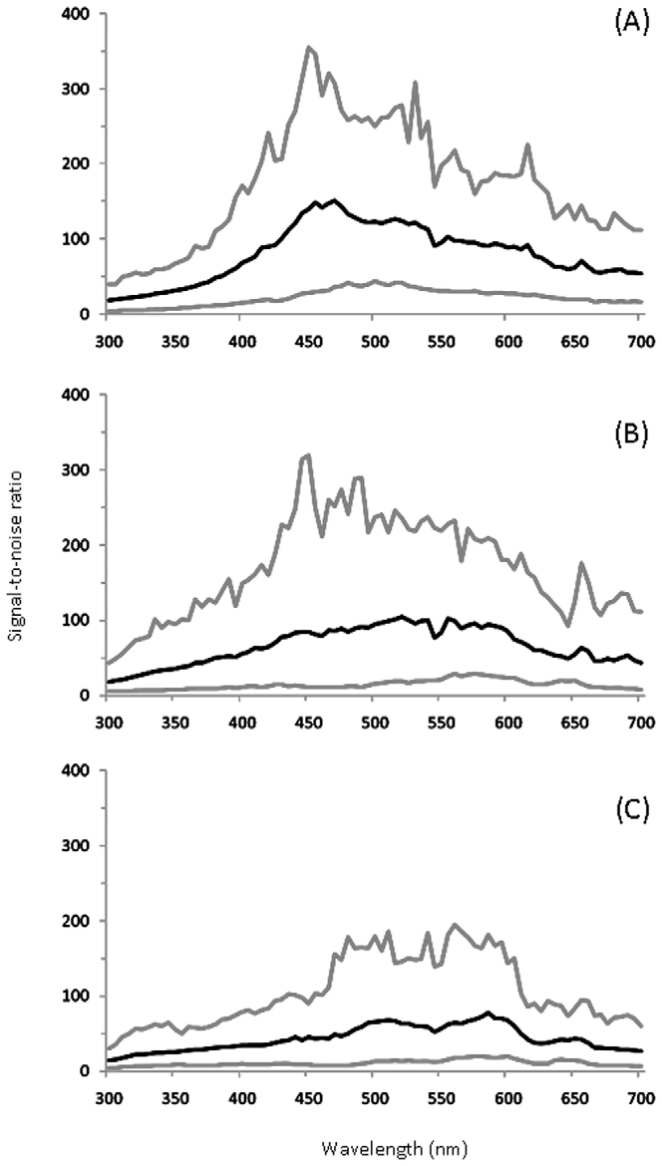
Signal-to-noise ratio for average reflectance spectra. Average (black line) and 5^th^ and 95^th^ percentiles (grey lines) at 5 nm steps across the wavelength, among the six replicate spectra across species with (A) immaculate, (B) partly maculated, and (C) heavily maculated eggshell types.

Over one-third of the species sampled (36.6%) had immaculate eggshells and we predicted that such eggshells may produce less variable replicate spectra within an egg. In order to determine whether measurement of background eggshell colour was more variable for maculated eggshells compared with immaculate eggshells, we compared the variability of reflectance spectra across immaculate eggshell types with maculated eggshell types (Figure 4). For a single randomly selected egg from each clutch, the signal-to-noise ratio (mean divided by the standard deviation) among the six replicate spectra within an egg was indeed greater, on average, for species with entirely immaculate eggs (Figure 4A) than species with maculated eggs (Figure 4B, 4C), Species with immaculate eggs also displayed a distinct maximum signal-to-noise ratio at 450 nm (Figure 4A).

**Figure 5:**
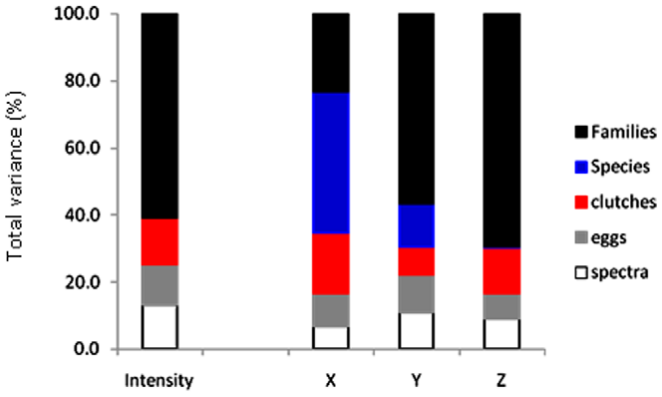
Taxonomic variability in perceived eggshell colour traits. Percentages of variability among eggshell reflectance measures from nested analysis of variance (nested ANOVA), for luminance (brightness) and each of the three independent tetrachromatic axes (X, Y, Z), that occur between replicate measures within an egg, between different eggs within a clutch, between different clutches within a species, between different species within a family, and between different families.

The Pagel's λ values for luminance and each of the three independent tetrachromatic axes (X, Y, Z) were all intermediate between 0.0 and 1.0, and significantly different from either (Table 1). This was true for both the equal branch length and proportional branch length phylogenies. Using a phylogenetic hypothesis constructed following Hackett et al. [40] (Text S1) the phylogenetic correlation is, in general, slightly higher, but our interpretation of the results remained unchanged (Table S1). Over 60% of the variability in luminance, among different spectra, occurred between families (Figure 5). The tetrachromatic axis with the greatest range of values was the Z-axis (range = 9.13) for which over 60% of the variability among spectra occurred between families (Figure 5).

**Table 1:** Phylogenetic correlations.

Equal branch length phylogeny, 1 df for all Likelihood-Ratio (LR) tests						
Level	Lambda (λ)	LnL λ	LnL1	LnL0	LR test 1	LR test 0
Luminance	0.798	−609.93	−624.12	−639.47	28.38	59.08
X	0.803	−345.83	−357.58	−377.52	23.50	63.38
Y	0.911	−327.76	−331.75	−371.60	7.98	87.68
Z	0.716	−465.67	−483.48	−484.44	35.62	37.54

Proportional branch length phylogeny, 1 df for all Likelihood-Ratio (LR) tests						
Level	Lambda (λ)	LnL λ	LnL1	LnL0	LR test 1	LR test 0
Luminance	0.827	−605.26	−671.45	−639.47	132.38	68.42
X	0.568	−346.32	−405.49	−377.52	118.34	62.40
Y	0.673	−332.12	−401.83	−371.60	139.42	78.96
Z	0.685	−467.05	−525.21	−484.44	116.32	34.78

Pagel's λ calculated for the four variables listed in the first column for both an equal branch length and proportional phylogenetic hypothesis (see Methods and Text S1). Pagel's λ is the degree of phylogenetic dependence of the data, calculated as the maximum likelihood estimate of the multiplier of the off-diagonal elements of the variance-covariance matrix implied by the phylogeny, following Freckleton et al. [44]. LnL λ is the log-likelihood of the maximum likelihood value of Pagel's λ. LnL1 is the log-likelihood value for the model with Pagel's λ set to 1. LnL0 is the log-likelihood value for the model with Pagel's λ set to 0 (equivalent to a standard general linear model). All maximum likelihood values of λ are significantly different from both 0 and 1, as calculated using a likelihood ratio test (α = 0.05).

## Discussion

Avian eggshell colours are varied and appear striking to human vision (Figure 1), yet the majority of bird species have background eggshell colours that are rather similar and overlap considerably in a predicted model of avian tetrachromatic colour space (Figure 1). Moreover, relative eggshell reflectance spectra can vary as much between closely related species (e.g. within genera) as they can between species from different families (Figure 2). Of particular interest are the differences in the percentages of taxonomic variability attributed to the different tetrachromatic axes (Figure 5). For example, the tetrachromatic axis along which the majority of the variability in eggshell colour aligned was the Z-axis (Figure 1A), which varies mostly among families, compared with the X-axis which varies considerably among species (within families). Whereas, the X-axis varies between species with differing contributions of ultraviolet reflectance, the Z-axis aligned (to the human eye) between red-brown and pure (eggshell) blue. For example, the largest difference along the Z-axis between the species in Figure 1C is between *Turdus philomelos* (a true thrush) and *Porzana tabuensis* (a crake) (ΔZ>9). Given that the Z-axis displayed the greatest range of values (Figure 1A), it is not surprising that the two tetrapyrrole pigments responsible for avian eggshell coloration [45]–[47] are a blue-green pigment (biliverdin) and a red-brown pigment (protoporphyrin). Both of these pigments are involved in the synthesis and catabolism of haem [48]–[50] and are both circulating in the bloodstream and metabolised de novo in the shell gland [51]–[53].

The region of greatest variability, in eggshell colour among closely related species, coincided with the medium-wavelength sensitive region around 500 nm. This region of the wavelength is most likely associated with differences in the presence (and concentration) of the bile-pigment biliverdin. Previously, it has been suggested that the production of either type of eggshell pigments is under independent genetic control [54], although both may be produced simultaneously (but in different concentrations) to generate the variety of perceived spectral differences in appearance. It is likely that key phylogenetic differences exist in the expression of these pigments and chemical analyses to support this will in all likelihood be greatly rewarding.

Our data are in broad agreement with previous analyses showing that differences in interspecific eggshell appearance is a relatively labile trait [6] and may not serve to aid the systematic ordering of birds because of strong underlying functional causes and adaptive roles of shell pigmentation and coloration [55]. However, our extensive taxonomic sampling also allows a number of novel, more specific conclusions. It is widely assumed, and there is no contrary evidence, that the ancestral avian egg was white (pigment free) and immaculate [3], [5]. Yet, both pigmentation and maculation are frequently expressed traits and, among the extant species we sampled, almost two-thirds had maculated eggs. Interestingly, the degree to which background eggshell coloration is evolutionarily conserved among species varies across the colour spectrum. For example, considerable variability existed between families at wavelengths that correspond with average peak spectral sensitivity for the ultra-short-, short- and long-wavelength sensitive regions. In contrast, for the intermediate (medium-wavelength sensitive) region most of the variation was at low taxonomic levels; between species within the same family (Figure 3).

The physical measurement and functional interpretation of colourful phenotypic traits (including eggshell appearance) has been greatly assisted by the use of portable reflectance spectrophotometers [14]. Subsequently, the analysis of reflectance-based data is a subject of considerable interest, and ongoing development, in studies of evolutionary [31], sensory [12], and behavioural biology [34]. Yet, it is not always clear how different measurements relate to different hypotheses of the adaptive function of coloration, or the life-history variability that underpins the pigments themselves. Previous studies of eggshell colour have not considered simultaneously differential selection across (avian) visible wavelengths. Similarly, the phylogenetic component to pigmentation at different wavelengths has not yet been addressed. We chose to analyse differences in reflectance across the entire avian visible spectrum, as well as through a representative, unbiased phylogenetic sampling protocol, to characterise where and how far from each other eggshell colours would lie in a hypothetical avian perceived colour space. This approach allowed us to interpret the differences among species without making any specific assumptions about how (or whether) these differences are perceived by the birds themselves, or their predators.

We do not find it particularly surprising that eggshell colours vary between species, even closely related ones. Many of the mechanisms proposed to drive egg colour diversity are associated with traits that are themselves labile at the species level, such as habitat use, nest site selection, sexual selection, brood parasitism, and predation pressure [21]. Signalling hypotheses, for example, propose that eggshell colour evolved from selective pressures associated with visual discrimination by the parental birds and/or predators. Such signalling hypotheses include: (1) avoiding predation; through either crypsis [56] or aposematism [57], (2) soliciting parental care [58], [59], (3) mimicry and/or crypsis of host eggs by brood parasites [60], [61], (4) facilitating own egg recognition as a strategy against intraspecific [62] and interspecific [63] brood parasitism, and (5) aiding the recognition of a parent's own egg(s) in dense breeding colonies [64]. Alternatively, structural hypotheses propose that eggshell colour evolved to enhance the physical protection of the developing embryo. Such structural hypotheses include: (1) combating harmful solar radiation [65], [66], (2) reinforcing eggshell strength [51], [67], (3) thermal regulation of the egg contents [68], and (4) antimicrobial defence [69], [70]. As previously noted [6], [9], analyses of eggshell coloration considering single functional hypotheses in isolation are insufficient. A broader comparative perspective is likely to be needed. In this context, future research on the adaptive function of eggshell pigmentation needs explicitly to account for our finding that related species can differ markedly in measures of background eggshell colour across different regions of the spectrum.

It is possible that our study under-estimates the diversity in eggshell appearance by only considering (1) a small proportion of all bird species (∼2.5%), and (2) only sampling from a single museum's collection. While our sampling is not biased with respect to overall avian phylogeny, adult body size, and geographic range, it is known that the properties of eggshell colour can be subject to environmental conditions [71] as well as changes (degradation) when they are stored in museum collections, rather than sampled from freshly laid eggs [22]. It is therefore of considerable interest to assess how eggshell colour changes with duration since collection and/or length of museum storage. In this regard, we detected significantly greater chromatic variability (but not luminance) across longer storage periods since collection. We note, however, that this effect is most likely to influence components of between clutch variability (within species) and that variance at this level is notably smaller than at most other sampling levels (Figure 3). We compared general traits that we assumed are more likely to respond equally across eggshells of different structural type and appearance. Our results support previous findings of temporal changes in museum collections [22] and we conclude that it remains important, whenever possible, to compare clutches of similar age to control for any inflation of colour variance among specimens. This will be particularly important for studies testing intraspecific hypotheses relating to adaptation of eggshell colour.

Overall, our analyses imply that divergent aspects of avian eggshell colour may be responding to selection from different evolutionary and/or ecological pressures. These pressures are variable at different levels of phylogenetic association in birds, and no single hypothesis is likely to be sufficient to explain the striking variation we observe in eggshell coloration. Consequently, our findings have significant implications for the interpretation of current species-specific, as well as more general, explanations for the evolution of eggshell pigmentation. The long-wavelength pigmentation, putatively involved in camouflage and thermoregulation [68], is more likely to be conserved at the family level, suggesting a general evolutionary advantage of this pigmentation. By contrast, medium-wavelength pigmentation varies as much between species as between families making it a candidate for more species specific adaptations, such as interactions between nest site selection and ecological behaviour [72]. Conversely, a small difference between closely related species in pigmentation at the longest or shortest wavelengths may indicate a more significant evolutionary adaptation than a much greater difference at medium wavelengths. The most rewarding question arising from the differential taxonomic variation in pigmentation is to what degree it is driven by ecological adaptation compared with phylogenetic differences in the physio-chemical production (or perception) of the different pigments. We look forward to further studies that attempt to unravel the phylogenetic association between the composition and concentration of eggshell pigments and the evolutionary drivers and functional impacts of variability in eggshell colour.

## Supporting Information

Figure S1 Putative avian phylogeny for the species sampled. Species for which average eggshell reflectance was greatest in the medium-wavelength sensitive region of the spectrum are coloured blue. Eggshells of all of the remaining species (coloured red) reflected maximally in the long-wavelength sensitive region.
(0.02 MB PDF)

Table S1 Phylogenetic correlations. Pagel's λ calculated for the same variables in Table 1 for phylogenetic hypotheses based on Hackett et al. [40] (see Methods and Text S1). All maximum likelihood values of λ are significantly different from both 0 and 1, with the exception of one (in bold), as calculated using a likelihood ratio test (α = 0.05).
(0.04 MB DOC)

Text S1 Details of the phylogeny used in our study.
(0.05 MB DOC)

Text S2 The phylogenetic hypothesis used in our study (Newick format).
(0.03 MB DOC)
