# Supplementary figures and images for: Variability in Avian Eggshell Colour: A Comparative Study of Museum Eggshells
Source: PLoS One. 2010 Aug 9;5(8):e12054. doi: 10.1371/journal.pone.0012054 (PMC2918502; doi:10.1371/journal.pone.0012054)

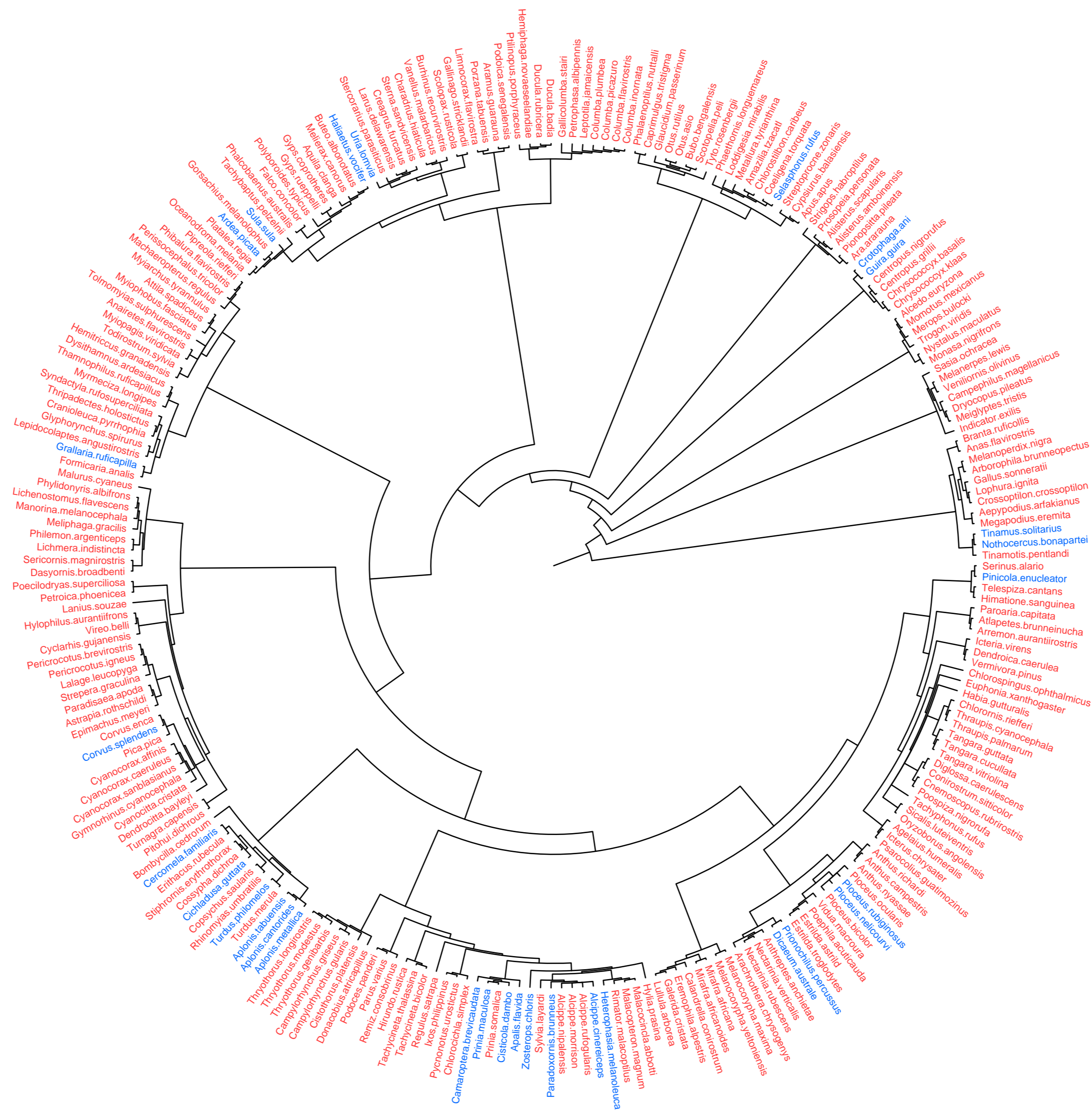

Supplement: Figure S1 — Putative avian phylogeny for the species sampled. Species for which average eggshell reflectance was greatest in the medium-wavelength sensitive region of the spectrum are coloured blue. Eggshells of all of the remaining species (coloured red) reflected maximally in the long-wavelength sensitive region. (0.02 MB PDF) [file pone.0012054.s001.pdf]
