# Supplementary material for: Variability in Avian Eggshell Colour: A Comparative Study of Museum Eggshells
Source: PLoS One. 2010 Aug 9;5(8):e12054. doi: 10.1371/journal.pone.0012054 (PMC2918502; doi:10.1371/journal.pone.0012054)
Supplement: Table S1 — Phylogenetic correlations. Pagel's λ calculated for the same variables in Table 1 for phylogenetic hypotheses based on Hackett et al. [40] (see Methods and Text S1). All maximum likelihood values of λ are significantly different from both 0 and 1, with the exception of one (in bold), as calculated using a likelihood ratio test (α = 0.05). (0.04 MB DOC) [file pone.0012054.s002.doc]

**Table S1 Phylogenetic correlations.** Pagel’s λ calculated for the same variables in Table 1 for phylogenetic hypotheses based on Hackett et al. [40] (see Methods and Text S1). All maximum likelihood values of λ are significantly different from both 0 and 1, with the exception of one (in bold), as calculated using a likelihood ratio test (α = 0.05).

| Equal branch length phylogeny, 1 df for all Likelihood-Ratio (LR) tests | | | | | | |
| --- | --- | --- | --- | --- | --- | --- |
| Level | Lambda (λ) | LnL λ | LnL1 | LnL0 | LR test 1 | LR test 0 |
| Luminance | 0.824 | -610.89 | -619.28 | -639.47 | 16.78 | 57.16 |
| X | 0.910 | -341.90 | -343.79 | -377.52 | **3.78** | 71.24 |
| Y | 0.931 | -331.32 | -333.80 | -371.60 | 4.96 | 80.56 |
| Z | 0.789 | -464.81 | -475.33 | -484.44 | 21.04 | 39.26 |
|  |  |  |  |  |  |  |
| Proportional branch length phylogeny, 1 df for all Likelihood-Ratio (LR) tests | | | | | | |
| Level | Lambda (λ) | LnL λ | LnL1 | LnL0 | LR test 1 | LR test 0 |
| Luminance | 0.683 | -596.18 | -681.06 | -639.47 | 169.76 | 86.58 |
| X | 0.498 | -344.63 | -414.93 | -377.53 | 140.60 | 65.80 |
| Y | 0.620 | -328.68 | -409.83 | -371.60 | 162.30 | 85.84 |
| Z | 0.655 | -457.61 | -531.18 | -484.44 | 147.14 | 53.66 |
