# Supplementary material for: Variability in Avian Eggshell Colour: A Comparative Study of Museum Eggshells
Source: PLoS One. 2010 Aug 9;5(8):e12054. doi: 10.1371/journal.pone.0012054 (PMC2918502; doi:10.1371/journal.pone.0012054)
Supplement: Text S1 — Details of the phylogeny used in our study. (0.05 MB DOC) [file pone.0012054.s003.doc]

**Text S1 Details of the phylogeny used in our study.** The phylogeny used for our analysis (Figure S1) was based on Sibley & Ahlquist ([1]), with some relationships at lower levels resolved using Jønsson & Fjeldså ([2]). The taxonomy of the same species is based on Sibley & Monroe ([3,4]). Many of the details of the Sibley & Ahlquist ([1]) phylogeny are highly contentious, but it remains the only comprehensive family-level bird phylogeny available for the taxa in our study. Moreover, we analyse egg colour evolution in terms of taxonomy as well as phylogeny, and the former approach makes many fewer assumptions about the branching order of subtaxa within taxa.

For the phylogenetic analysis, we assumed that branch lengths were proportional to the number of species below the node [5]. We made this assumption because true branch lengths are unknown. However, we have shown in the context of previous analyses [6] that branch lengths calculated this way produce node ages that are highly correlated with the DT50H values provided by Sibley & Ahlquist ([1]) for their phylogeny (r = 0.93, n = 20 nodes). Thus, our branch length assumption produces a phylogeny with a distribution of node ages that conforms well to quantitative data. Branch lengths were calculated using TreeEdit v. 1.0. The phylogeny is presented in Newick format (Text S2).

We repeated the phylogenetic correlation (Table 1 c.f. Table S1) using a phylogeny with relationships amongst orders based on Hackett et al. ([7], their figures 2 and 4). Because Hackett et al. provide little detail of relationships below orders, we assumed (i) that oscines and suboscines are sister taxa, (ii) that corvida and passerida (sensu [3]) are sister taxa, (iii) that the phylogeny of the Passerida follows Ericson & Johansson ([8]), (iv) that Malurus is an outgroup to other oscine passerines, and (v) that relationships within families follow Sibley & Ahlquist ([1]). In fact the majority of families in our analysis are represented by few species, with only the Fringillidae (sensu [8]) represented by more than 10. A copy of this phylogeny is also available on request.

**References**

1. Sibley, CG, Ahlquist, JE (1990) Phylogeny and classification of birds: a study in molecular evolution. New Haven, USA: Yale University Press. 1080 p.

2. Jønsson, KA, Fjeldså, J (2006) A phylogenetic supertree of oscine passerine birds (Aves: Passeri). Zoo Scrip 35: 149-186.

3. Sibley, CG, Monroe, BL Jr (1990) Distribution and taxonomy of birds of the world. New Haven, USA: Yale University Press. 1136 p.

4. Sibley, CG, Monroe, BL Jr (1993) Supplement to the distribution and taxonomy of birds of the world. New Haven, USA: Yale University Press. 108 p.

5. Grafen, A. (1989) The phylogenetic regression. Phil Trans R Soc Lond B 326: 119-157.

6. Halsey, LG, Butler, PJ, Blackburn, TM (2006) A phylogenetic analysis of the allometry of diving. Am Nat 167: 276-287.

7. Hackett SJ, Kimball RT, Reddy S, Bowie RCK, Braun EL et al. (2008) A phylogenomic study of birds reveals their evolutionary history. Science 320: 1763-1768.

8. Ericson, PGP, Johansson, U.S. (2003) Phylogeny of Passerida (Aves: Passeriformes) based on nuclear and mitochondrial sequence data. Mol Phyl Evol 29: 126-138.
