# Supplementary material for: Variability in Avian Eggshell Colour: A Comparative Study of Museum Eggshells
Source: PLoS One. 2010 Aug 9;5(8):e12054. doi: 10.1371/journal.pone.0012054 (PMC2918502; doi:10.1371/journal.pone.0012054)
Supplement: Text S2 — The phylogenetic hypothesis used in our study (Newick format). (0.03 MB DOC) [file pone.0012054.s004.doc]

**Text S2 The phylogenetic hypothesis used in our study (Newick format).**

(((((((((((((((('Serinus.alario':1.00000000,'Pinicola.enucleator':1.00000000):2.00000000,('Telespiza.cantans':1.00000000,'Himatione.sanguinea':1.00000000):2.00000000):25.00000000,(('Paroaria.capitata':2.00000000,('Atlapetes.brunneinucha':1.00000000,'Arremon.aurantiirostris':1.00000000):1.00000000):22.00000000,(('Icteria.virens':2.00000000,('Dendroica.caerulea':1.00000000,'Vermivora.pinus':1.00000000):1.00000000):19.00000000,(('Chlorospingus.ophthalmicus':15.00000000,('Euphonia.xanthogaster':14.00000000,('Habia.gutturalis':13.00000000,(('Chlorornis.riefferi':2.00000000,('Thraupis.cyanocephala':1.00000000,'Thraupis.palmarum':1.00000000):1.00000000):3.00000000,('Tangara.guttata':2.00000000,('Tangara.cucullata':1.00000000,'Tangara.vitriolina':1.00000000):1.00000000):3.00000000):8.00000000,(('Diglossa.caerulescens':1.00000000,'Conirostrum.sitticolor':1.00000000):2.00000000,('Cnemoscopus.rubrirostris':1.00000000,'Poospiza.nigrorufa':1.00000000):2.00000000):10.00000000,'Tachyphonus.rufus':13.00000000,('Sicalis.luteiventris':1.00000000,'Oryzoborus.angolensis':1.00000000):12.00000000):1.00000000):1.00000000):3.00000000,('Agelaius.humeralis':2.00000000,('Icterus.chrysater':1.00000000,'Psarocolius.guatimozinus':1.00000000):1.00000000):16.00000000):3.00000000):3.00000000):4.00000000):17.00000000,((('Anthus.richardi':2.00000000,('Anthus.campestris':1.00000000,'Anthus.nyassae':1.00000000):1.00000000):8.00000000,(('Ploceus.ocularis':3.00000000,'Ploceus.rubiginosus':3.00000000,'Ploceus.nelicourvi':3.00000000,'Ploceus.bicolor':3.00000000):4.00000000,('Vidua.macroura':3.00000000,('Poephila.acuticauda':2.00000000,('Estrilda.astrild':1.00000000,'Estrilda.troglodytes':1.00000000):1.00000000):1.00000000):4.00000000):3.00000000):6.00000000,(('Prionochilus.percussus':1.00000000,'Dicaeum.australe':1.00000000):4.00000000,(('Anthreptes.anchietae':2.00000000,('Nectarinia.verticalis':1.00000000,'Nectarinia.rubescens':1.00000000):1.00000000):1.00000000,'Arachnothera.chrysogenys':3.00000000):2.00000000):11.00000000):29.00000000):8.00000000,(('Melanocorypha.maxima':1.00000000,'Melanocorypha.yeltoniensis':1.00000000):6.00000000,(('Mirafra.africana':1.00000000,'Mirafra.africanoides':1.00000000):4.00000000,('Calandrella.conirostrum':3.00000000,('Eremophila.alpestris':2.00000000,('Galerida.cristata':1.00000000,'Lullula.arborea':1.00000000):1.00000000):1.00000000):2.00000000):2.00000000):46.00000000):34.00000000,(((((((('Hylia.prasina':10.00000000,(((('Malacocincla.abbotti':1.00000000,'Malacopteron.magnum':1.00000000):1.00000000,'Rimator.malacoptilus':2.00000000):1.00000000,'Heterophasia.melanoleuca':3.00000000):6.00000000,((('Alcippe.cinereiceps':3.00000000,'Alcippe.rufogularis':3.00000000,'Alcippe.morrison':3.00000000,'Alcippe.nipalensis':3.00000000):1.00000000,'Paradoxornis.brunneus':4.00000000):1.00000000,'Sylvia.layardi':5.00000000):4.00000000):1.00000000):1.00000000,'Zosterops.chloris':11.00000000):5.00000000,('Apalis.flavida':4.00000000,(('Cisticola.dambo':2.00000000,('Prinia.somalica':1.00000000,'Prinia.maculosa':1.00000000):1.00000000):1.00000000,'Camaroptera.brevicaudata':3.00000000):1.00000000):12.00000000):3.00000000,('Chlorocichla.simplex':2.00000000,('Pycnonotus.urostictus':1.00000000,'Ixos.philippinus':1.00000000):1.00000000):17.00000000):1.00000000,'Regulus.satrapa':20.00000000):3.00000000,(('Tachycineta.bicolor':1.00000000,'Tachycineta.thalassina':1.00000000):1.00000000,'Hirundo.rustica':2.00000000):21.00000000):3.00000000,('Remiz.consobrinus':2.00000000,('Parus.varius':1.00000000,'Podoces.panderi':1.00000000):1.00000000):24.00000000):7.00000000,('Donacobius.atricapillus':6.00000000,('Cistothorus.platensis':5.00000000,(('Campylorhynchus.gularis':1.00000000,'Campylorhynchus.griseus':1.00000000):3.00000000,('Thryothorus.genibarbis':2.00000000,('Thryothorus.modestus':1.00000000,'Thryothorus.longirostris':1.00000000):1.00000000):2.00000000):1.00000000):1.00000000):27.00000000):54.00000000):13.00000000,((('Aplonis.metallica':2.00000000,('Aplonis.cantorides':1.00000000,'Aplonis.tabuensis':1.00000000):1.00000000):9.00000000,(('Turdus.merula':1.00000000,'Turdus.philomelos':1.00000000):7.00000000,(('Rhinomyias.umbratilis':1.00000000,'Copsychus.saularis':1.00000000):5.00000000,(('Cichladusa.guttata':2.00000000,('Cossypha.dichroa':1.00000000,'Stiphrornis.erythrothorax':1.00000000):1.00000000):2.00000000,('Erithacus.rubecula':1.00000000,'Cercomela.familiaris':1.00000000):3.00000000):2.00000000):2.00000000):3.00000000):1.00000000,'Bombycilla.cedrorum':12.00000000):88.00000000):33.00000000,(((((('Pitohui.dichrous':1.00000000,'Turnagra.capensis':1.00000000):16.00000000,(('Dendrocitta.bayleyi':8.00000000,(('Cyanocitta.cristata':4.00000000,('Gymnorhinus.cyanocephala':3.00000000,('Cyanocorax.sanblasianus':2.00000000,'Cyanocorax.caeruleus':2.00000000,'Cyanocorax.affinis':2.00000000):1.00000000):1.00000000):3.00000000,('Pica.pica':2.00000000,('Corvus.splendens':1.00000000,'Corvus.enca':1.00000000):1.00000000):5.00000000):1.00000000):7.00000000,(('Epimachus.meyeri':2.00000000,('Astrapia.rothschildi':1.00000000,'Paradisaea.apoda':1.00000000):1.00000000):4.00000000,('Strepera.graculina':3.00000000,('Lalage.leucopyga':2.00000000,('Pericrocotus.igneus':1.00000000,'Pericrocotus.brevirostris':1.00000000):1.00000000):1.00000000):3.00000000):9.00000000):2.00000000):3.00000000,('Cyclarhis.gujanensis':2.00000000,('Vireo.belli':1.00000000,'Hylophilus.aurantiifrons':1.00000000):1.00000000):18.00000000):1.00000000,'Lanius.souzae':21.00000000):2.00000000,('Petroica.phoenicea':1.00000000,'Poecilodryas.superciliosa':1.00000000):22.00000000):9.00000000,((('Dasyornis.broadbenti':1.00000000,'Sericornis.magnirostris':1.00000000):6.00000000,(('Lichmera.indistincta':1.00000000,'Philemon.argenticeps':1.00000000):4.00000000,('Meliphaga.gracilis':3.00000000,('Manorina.melanocephala':2.00000000,('Lichenostomus.flavescens':1.00000000,'Phylidonyris.albifrons':1.00000000):1.00000000):1.00000000):2.00000000):2.00000000):1.00000000,'Malurus.cyaneus':8.00000000):24.00000000):101.00000000):22.00000000,(((('Formicaria.analis':1.00000000,'Grallaria.ruficapilla':1.00000000):5.00000000,(('Lepidocolaptes.angustirostris':1.00000000,'Glyphorynchus.spirurus':1.00000000):3.00000000,(('Cranioleuca.pyrrhophia':1.00000000,'Thripadectes.holostictus':1.00000000):1.00000000,'Syndactyla.rufosuperciliata':2.00000000):2.00000000):2.00000000):3.00000000,('Myrmeciza.longipes':2.00000000,('Thamnophilus.ruficapillus':1.00000000,'Dysithamnus.ardesiacus':1.00000000):1.00000000):7.00000000):12.00000000,((('Hemitriccus.granadensis':1.00000000,'Todirostrum.sylvia':1.00000000):6.00000000,('Myiopagis.viridicata':1.00000000,'Anairetes.flavirostris':1.00000000):6.00000000,'Tolmomyias.sulphurescens':7.00000000,('Myiophobus.fasciatus':2.00000000,('Attila.spadiceus':1.00000000,'Myiarchus.tyrannulus':1.00000000):1.00000000):5.00000000):4.00000000,('Machaeropterus.regulus':3.00000000,('Perissocephalus.tricolor':2.00000000,('Phibalura.flavirostris':1.00000000,'Pipreola.riefferi':1.00000000):1.00000000):1.00000000):8.00000000):10.00000000):134.00000000):40.00000000,(((((((('Oceanodroma.melania':1.00000000,'Platalea.regia':1.00000000):2.00000000,('Ardea.picata':1.00000000,'Gorsachius.melanolophus':1.00000000):2.00000000):1.00000000,'Sula.sula':4.00000000):1.00000000,'Tachybaptus.pelzelnii':5.00000000):9.00000000,(('Phalcobaenus.australis':1.00000000,'Falco.concolor':1.00000000):7.00000000,('Polyboroides.typicus':6.00000000,(('Gyps.rueppelli':1.00000000,'Gyps.coprotheres':1.00000000):4.00000000,('Aquila.clanga':3.00000000,('Melierax.canorus':2.00000000,('Buteo.albonotatus':1.00000000,'Haliaetus.vocifer':1.00000000):1.00000000):1.00000000):2.00000000):1.00000000):2.00000000):6.00000000):10.00000000,((('Uria.lomvia':4.00000000,('Stercorarius.parasiticus':3.00000000,(('Larus.delawarensis':1.00000000,'Creagrus.furcatus':1.00000000):1.00000000,'Sterna.sandvicensis':2.00000000):1.00000000):1.00000000):3.00000000,(('Charadrius.hiaticula':1.00000000,'Vanellus.malarbaricus':1.00000000):1.00000000,'Burhinus.recurvirostris':2.00000000):5.00000000):2.00000000,('Scolopax.rusticola':1.00000000,'Gallinago.stricklandii':1.00000000):8.00000000):15.00000000):4.00000000,(('Limnocorax.flavirostra':1.00000000,'Porzana.tabuensis':1.00000000):2.00000000,('Aramus.guarauna':1.00000000,'Podoica.senegalensis':1.00000000):2.00000000):25.00000000):11.00000000,(('Ptilinopus.porphyraceus':3.00000000,('Hemiphaga.novaeseelandiae':2.00000000,('Ducula.rubricera':1.00000000,'Ducula.badia':1.00000000):1.00000000):1.00000000):7.00000000,('Gallicolumba.stairi':6.00000000,('Petrophasa.albipennis':5.00000000,('Leptotila.jamaicensis':4.00000000,('Columba.plumbea':3.00000000,('Columba.picazuro':2.00000000,('Columba.flavirostris':1.00000000,'Columba.inornata':1.00000000):1.00000000):1.00000000):1.00000000):1.00000000):1.00000000):4.00000000):29.00000000):156.00000000):18.00000000,((('Phalaenoptilus.nuttalli':1.00000000,'Caprimulgus.tristigma':1.00000000):6.00000000,(('Glaucidium.passerinum':4.00000000,(('Otus.rutilus':1.00000000,'Otus.asio':1.00000000):2.00000000,('Bubo.bengalensis':1.00000000,'Scotopelia.peli':1.00000000):2.00000000):1.00000000):1.00000000,'Tyto.rosenbergii':5.00000000):2.00000000):10.00000000,(('Phaethornis.longuemareus':6.00000000,('Loddigesia.mirabilis':5.00000000,('Metallura.tyrianthina':4.00000000,(('Amazilia.tzacatl':1.00000000,'Chlorostilbon.caribeus':1.00000000):2.00000000,('Coeligena.torquata':1.00000000,'Selasphorus.rufus':1.00000000):2.00000000):1.00000000):1.00000000):1.00000000):3.00000000,('Streptoprocne.zonaris':2.00000000,('Cypsiurus.balasiensis':1.00000000,'Apus.apus':1.00000000):1.00000000):7.00000000):8.00000000):196.00000000):6.00000000,('Strigops.habroptilus':5.00000000,(('Prosopeia.personata':2.00000000,('Alisterus.scapularis':1.00000000,'Alisterus.amboinensis':1.00000000):1.00000000):2.00000000,('Pionopsitta.pileata':1.00000000,'Ara.ararauna':1.00000000):3.00000000):1.00000000):214.00000000):6.00000000,(('Crotophaga.ani':1.00000000,'Guira.guira':1.00000000):4.00000000,(('Centropus.nigrorufus':1.00000000,'Centropus.grillii':1.00000000):2.00000000,('Chrysococcyx.basalis':1.00000000,'Chrysococcyx.klaas':1.00000000):2.00000000):2.00000000):220.00000000):6.00000000,(((('Alcedo.euryzona':1.00000000,'Momotus.mexicanus':1.00000000):1.00000000,'Merops.bulocki':2.00000000):1.00000000,'Trogon.viridis':3.00000000):2.00000000,('Nystalus.maculatus':1.00000000,'Monasa.nigrifrons':1.00000000):4.00000000):226.00000000):7.00000000,(('Sasia.ochracea':5.00000000,(('Melanerpes.lewis':1.00000000,'Veniliornis.olivinus':1.00000000):3.00000000,('Campephilus.magellanicus':2.00000000,('Dryocopus.pileatus':1.00000000,'Meiglyptes.tristis':1.00000000):1.00000000):2.00000000):1.00000000):1.00000000,'Indicator.exilis':6.00000000):232.00000000):12.00000000,((('Branta.ruficollis':1.00000000,'Anas.flavirostris':1.00000000):7.00000000,((('Melanoperdix.nigra':1.00000000,'Arborophila.brunneopectus':1.00000000):3.00000000,('Gallus.sonneratii':2.00000000,('Lophura.ignita':1.00000000,'Crossoptilon.crossoptilon':1.00000000):1.00000000):2.00000000):2.00000000,('Aepypodius.arfakianus':1.00000000,'Megapodius.eremita':1.00000000):5.00000000):2.00000000):3.00000000,(('Tinamus.solitarius':1.00000000,'Nothocercus.bonapartei':1.00000000):1.00000000,'Tinamotis.pentlandi':2.00000000):9.00000000):239.00000000);
